# Relationship between Health Literacy and Sexual Function and Sexual Satisfaction in Infertile Couples Referred to The Royan Institute 

**DOI:** 10.22074/ijfs.2018.5185

**Published:** 2018-03-18

**Authors:** Mohammad Sahebalzamani, Zahra Mostaedi, Hojjatollah Farahani, Mobin Sokhanvar

**Affiliations:** 1 Department of Management, Tehran Medical Sciences Branch Islamic Azad University, Tehran, Iran; 2 Department of Nursing and Midwifery, Tehran Medical Sciences Branch Islamic Azad University, Tehran, Iran; 3 Department of Psychology, Tehran Medical Sciences Branch Islamic Azad University, Tehran, Iran; 4 Iranian Center of Excellence in Health Management (IceHM), Tabriz University of Medical Sciences, Tabriz, Iran

**Keywords:** Health Literacy, Infertility, Sexual Dysfunction, Sexual Satisfaction

## Abstract

**Background:**

Health science and technology today is a rapidly growing field. Health is a multifaceted concept influ- enced by several factors, and health literacy is essential to deal properly with the current situation. In this study, the association between health literacy and sexual function and sexual satisfaction were investigated in 2016.

**Materials and Methods:**

This descriptive and correlational study was conducted on 193 couples in the Royan Insti- tute, Tehran. Data collection instruments were three standard questionnaires which included the Test of Functional Health Literacy, the Female Sexual Function Index (FSFI) and the International Index of Erectile Function, and the Iranian version of the Sexual Satisfaction Scale. The data were analyzed using SPSS-v23 software at a significance level of 0.05.

**Results:**

Marginal health literacy, 49.7% among men and 44.1% among women, was more common than adequate or inadequate health literacy. Erectile function for the majority of men was appropriate (53.3%), compared to 16.6% who had perfect function and 30.1% for whom function was less than appropriate. The majority of women (57.0%) had sexual dysfunction. One hundred and three (53.3%) men had appropriate sexual function and 57% of women had normal sexual function. The greater proportion of men (50.8%) and women (46.1%) had good, rather than very good or less than good, sexual satisfaction. The results of chi-square tests indicated that greater health literacy was associ- ated with higher levels of sexual function and sexual satisfaction among men and women. However, application of the Cramer’s V test indicates that the strength of these associations is moderate to weak.

**Conclusion:**

Health literacy was marginal among most couples and its adverse impacts on sexual function and sexual satisfaction were confirmed. Accordingly, it is recommended that plans be developed to promote health literacy among infertile couples.

## Introduction

Health literacy represents the capacity of a person to access, interpret, and apply health information as well as make decisions to use existing health services (1). This concept was used for the first time in the field of health education in 1974. In the first two decades after the development of this concept, little attention was paid to it. However, once it started to be used formally in health promotion texts from 1997, attention to the concept has increased (2). Health literacy is one of the components that determines an individual’s health-related behaviors and likelihood of following treatment recommendations. Effective decisions about health are also affected by health literacy, and general health and the adoption of preventive behaviors depend on it (3, 4).

Several studies have been conducted to determine the effects of health literacy on different health outcomes. In this regard, we can refer to the impact of health literacy on quality of life, medication and adherence to clinical recommendations, taking advantage of contraceptive and emergency services, reducing the risk of mortality, utilization of services and knowledge related to asthma, the use of mammography and effective use of health information sources (5-11). This in turn can affect the quantity and quality of an individual’s sexual relations and sexual satisfaction.

Good sexual function between couples includes regu. lar willingness to participate in sexual acts, sexual arous. al, and reaching orgasm and increase their marital satisfaction (12). Sexual satisfaction is dependent on mental state as well as attitudes to sexual relations and the level of pleasure derived (13). Sexual function and satisfaction also have physical and psychological dimensions. Sexual functioning and the quality of that functioning are affected not only by an individual’s physical condition, but also by their mental state. Indeed a person’s mental state may be the main component in determining sexual satisfaction (14). In this regard, results of a study conducted on 176 women with cervical cancer and a history of pelvic radiation therapy showed one third of them had sexual dysfunction due to their unfavorable physical condition and consequently psychological problems (15). Therefore, we can conceptually explain the relationship between health literacy and sexual satisfaction and function. Health literacy affects the behavior of individuals, how they make use of information and services, and their physical and mental condition (16). These variables affect people’s sexual function and satisfaction.

Studies of health literacy among pregnant women in Tehran showed literacy to be adequate in 45.4%. The proportion of people with marginal and inadequate health literacy was respectively 24.6 and 30% (3). In addition, results of a study which conducted on 525 adults in Isfahan showed that 46.5 percent of people had adequate health literacy (17). The results of a study of 130 infertile women referred to the Montaserieh Infertility Research Centre in Mashhad showed that 54.6 had inadequate or poor sexual function (18). Investigation of sexual function among women referred to health centers in north Tehran showed the prevalence of sexual dysfunction to be 64% and the level of sexual satisfaction only 20% (19). The results of a study conducted in Turkey showed sexual dissatisfaction in 102 infertile couples to be 37.3 and 33.3% for women and men respectively (20).

Health literacy is one of the factors that affect health behaviors and several aspects of health. These include sexual function and sexual satisfaction, which in turn affect quality of life in terms marital satisfaction. Therefore, in this study, the relationship between health literacy and sexual function and sexual satisfaction was investigated in infertile couples referred to the Royan Institute in 2016.

## Materials and Methods

In this descriptive study conducted in 2016 health literacy, sexual function and sexual satisfaction and correlations between them were examined. The research field was Royan Institute in Tehran and the research population included all of the couples (with primary and secondary infertility) referred to the Institute during the study. The Cochran formula was used to calculate the sample size (z=1.96, a=0.05, p=0.5, q=0.5, and d=0.05). A sample of 386 people was selected to give the ability to detect differences with a significance level of 0.05 and a power of 80%.

The sample of 193 women and 193 men was selected using availability sampling by researchers who visited the Institute on specific days. Participants of the study included couples who lived together and were both willing to participate in the study. They also had either a diagnosis of primary infertility (no pregnancy for 12 months despite marital relations without contraception and no history of previous pregnancy) or a diagnosis of secondary infertility (no pregnancy despite a history of previous pregnancy) (21). In order to obtain the required data, four valid and reliable questionnaires were administered.

These included a demographic characteristics questionnaire, the Test of Functional Health Literacy in Adults (TOFHLA), a standard sexual functioning questionnaire (separate versions for women and men) and the Iranian version of the sexual satisfaction questionnaire (separate versions for women and men). The first questionnaire collected information on age, education, housing, household income, age at marriage, occupation, disease and habit (cigarette, alcohol, and drug) history of each individual and their relatives, marriage duration, weight, height, number of children, pregnancy history, abortion experience, treatment seeking, diagnosis, infertility cause, and contraception method. The TOFHLA questionnaire of Parker et al. (22) has two components, one for reading and one for numeracy, together comprised of 67 questions. Overall score for this tool was 100, with a score of 0-59 representing inadequate health literacy, 60-74 marginal health literacy, and 75-100 adequate health literacy.

Face validity, content validity and reliability of this tool (translated version) were studied and verified in the study conducted by Javadzade et al. (23) (reading component- Cronbach’s alpha=0.88, numeracy component Cronbach’s alpha=0.79). The 19-item Female Sexual Function Index (FSFI) of Rosen et al. (24) was used to assess sexual function in women. It covers six functional areas including desire, arousal, vaginal moisture, orgasm, pain, and sexual satisfaction. The numerical range for these fields is defined from 1 to 5. Total score less than 19 indicates the presence of sexual dysfunction. Karamidehkordi and Roudsari (18) confirmed the validity of the Persian version of FSFI, it was also confirmed its (Cronbach’s alpha= 0.79).

To measure sexual function in men, the International Index of Erectile Function (IIEF) was used. The questionnaire, developed by Rosen et al, includes five dimensions of erectile function, ejaculatory function, sexual stimulation, satisfaction during intercourse, and overall satisfaction in 15 questions. Each items are scored from 0 to 5, or 1 to 5, in which higher scores indicate better sexual performance (25). Content validity of the instrument (Persian version) was confirmed in a study conducted by Fakhri et al. (26) and reliability was confirmed by Cronbach’s alpha. A self-assessment tool, developed in a study by Nasiri Nejad et al. (27), was used to measure sexual satisfaction. The total score for this tool was 108 for men and 144 for women. Low, moderate, good, and very good satisfaction levels were categorized by scores that differ by increments of 27 in men and 36 in women. Face validity, content validity and reliability of this tool (Cronbach’s alpha 0.89for the male questionnaire and 0.859 for the female questionnaire) were confirmed. Data were collected by interview after explaining the purpose of the research to the participants and obtaining informed consent.

### Ethical approval

The study was designed in accordance with ethical rules approved by the Ethical Committee of the Islamic Azad University of Tehran Medical branch (IR.IAU.TMU. REC.1394.23).

### Statistical analysis

The data collected in this study were analyzed using SPSS 23.0 statistical software (SPSS, Inc., Chicago, IL, USA). Descriptive statistics including percent’s and frequencies are reported. Chi-square tests were used to analyze the associations between sexual function or sexual satisfaction and heath literacy at significant level of 0.05. Although the chi-squared test can indicate whether or not there is a relationship between 2 qualitative variables, it cannot indicate the strength of the association. The Cramer’s V test was thus used to determine the strength of the associations and, consequently, their importance. 

## Results

Demographic variables are shown in Table 1. Besides that, about 64% of the participants weighed 60 kg or above, and the height of the majority (48.7%) was in the range of 150-170 cm. Although 86% of subjects had no children, only 10.6% had a history of non-pregnancy among family members and close relatives. Additionally, 85.5% of participants had no history of previous pregnancy and the percentage that had experienced an abortion was estimated 3.1%. For 63.2% of the participants this was their first referral to a health center for follow up of their infertility status, and only 40.4% of them had a diagnosis of the cause of their infertility. Twenty eight percent of the 40.4% infertility was due to the woman, and 59.1% of the participants used medicinal methods of contraception.

Descriptive results related to health literacy, sexual function, and sexual satisfaction variables are shown in Table 2. The results reported in Table 2 show that a marginal level of health literacy (44.1% in women and 49.7% in men) was more common that an adequate or inadequate level. In both sexes the percentages of participants with an inadequate level of health literacy was lower than those with an adequate level. In addition, 57.0% of women had normal sexual function and 69.9% of men had appropriate or perfect sexual function. The percentage of participants with good or very good sexual satisfaction was 57.0% in women and 67.4% in men.

**Table 1:** demographic variables for infertile couples referred to the Royan Institute in 2016


Variable	Subgroup	Frequency (%)	Variable	Subgroup	Frequency (%)

Age (Y)	<20	9 (2.4)	Marriage age (Y)	<20	41 (10.6)
	20-30	143 (37.0)		20-30	265 (68.7)
	31-40	173 (44.8)		31-40	61 (15.8)
	>40	61 (15.8)		>40	19 (4.9)
	Total	386 (100)		Total	386 (100)
Education	Illiterate	5 (1.3)	Occupation	Housewife	27 (7.0)
	Primary	56 (14.5)		Worker	88 (22.8)
	≤Diploma	163 (42.3)		Employee	99 (25.7)
	Bachelor	133 (34.4)		Self-Employed	160 (41.4)
	>Bachelor	29 (7.5)		Unemployed	12 (3.1)
	Total	386 (100)		Total	386 (100)
Housing	Owner- Occupied	108 (28.0)	History: individual (I), relatives (R)	Special diseases (I&R)	17 (12.0)
	Rented	218 (56.5)		Chronic diseases (I)	38 (27.0)
	Corporate Home	42 (10.9)		Genetic diseases (I&R)	13 (9.2)
	Relative’s	18 (4.6)		Cigarette, alcohol, and drug (I)	73 (51.8)
	Total	386 (100)		Total	141 (100)
Household income (Million Rial)	<1	6 (1.5)	Marriage duration (Y)	<1	8 (2)
	1-2	154 (39.9)		1-5	152 (39.4)
	2-3	174 (45.1)		6-10	174 (45.1)
	>3	52 (13.5)		>10	52 (13.5)
	Total	386(100)		Total	386(100)


**Table 2:** Health literacy, sexual function, and sexual satisfaction in infertile couples referred to the Royan Institute in 2016


Variable	Sample	Level of variable	Frequency (%)

Health literacy	Woman	Inadequate	46 (23.8)
		Marginal	85 (44.1)
		Adequate	62 (32.1)
		Total	193 (100)
	Man	Inadequate	42 (21.8)
		Marginal	96 (49.7)
		Adequate	55 (28.5)
		Total	193 (100)
Sexual function	Woman	Dysfunction	83 (43.1)
		Normal	110 (57.1)
		Total	193 (100)
	Man	Inappropriate	3 (1.6)
		Medium	11 (5.7)
		Medium to Good	44 (22.8)
		Appropriate	103 (53.3)
		Perfect	32 (16.6)
		Total	193 (100)
Sexual satisfaction	Woman	Low	27 (14.0)
		Medium	56 (29.0)
		Good	89 (46.1)
		Very Good	21 (10.9)
		Total	193 (100)
	Man	Low	17 (8.8)
		Medium	46 (23.8)
		Good	98 (50.8)
		Very Good	32 (16.6)
		Total	193 (100)


The relationships between health literacy, sexual function, and sexual satisfaction were examined using the chi-square test and are presented in Table 3. The results show a significant relationship (P<0.05) between the level of health literacy, sexual function, and sexual satisfaction in infertile women and men referred to the Royan Institute. Cramer's V indicated that the strength of the association between health literacy and sexual function in women is 0.33 and 0.18 for men. The Cramer’s V results indicate that these relationships can be considered as moderate to weak.

## Discussion

Marginal health literacy, 49.7% among men and 44.1% among women, was more common than adequate or inadequate health literacy. Ghanbari et al. (3) in their study of fertile women concluded that 45.4% had an adequate literacy level and the percentage with a marginal literacy level was only 24.6%. Results of a study of 525 adults in Isfahan city showed that 46.5% had adequate health literacy and the proportion with marginal and inadequate literacy was 38.0 and 15.5%, respectively (17). Another study conducted by Protheroe et al. (28) in an English city showed that 28.5% of adult papulation had inadequate health literacy levels. However, the results of a study conducted among American adults showed that half the participants do not have basic health literacy (29). In another study conducted among African-American adults, it was shown that 65% of participants had low and inadequate health literacy levels (30). Due to the dependency of health literacy on socio-economic and cultural conditions, the difference between our results whit other studies which conducted in different regions is natural. 

In relation to sexual functioning, our study showed 43 percent of the women had sexual dysfunction and 53.3% of men had appropriate sexual function. Results of other studies in Iran are not in line whit our study. In the North of Tehran, the prevalence of sexual dysfunction among women was 64% (19). Another study conducted on 405 women in South Tehran showed that sexual function in the largest proportion was moderate (31). The study of Karamidehkordi and Roudsari (18) on 130 infertile women in Mashhad showed that 45.4% of infertile women had normal sexual function.

Cai et al. (32) conducted a study of 105 infertile women in China which showed the mean sexual function score in infertile women was 25.2 (out of 28). In Turkey, sexual dysfunction in women affected by male infertility was 51.9%, while it was 54.8% in the group affected by female infertility (33). The results of a study conducted on 236 men in South Korea (sex partners of infertile couples) showed that only 49.2% did not suffer sexual dysfunction. The remaining 50.8% had different levels of sexual dysfunction, a rate that is high in comparison with the present study (34). In addition, a study conducted in Turkey on 56 men showed 85.9% of participants had mild to moderate sexual dysfunction (35). 

The third variable examined in this study was sexual satisfaction, and, based on the findings, 57% of women and 67.4% of men had good or very good sexual satisfaction. Ramazani et al. (19) concluded that 20% of participants had sexual satisfaction with their spouse. In south of Tehran, a significant percentage of women (58.2%) had moderate sexual satisfaction level (31). A study conducted in Egypt on infertile couples (due to male infertility) showed that 89% of men and 80% of women had sexual satisfaction with their spouses (36). The difference in sexual satisfaction found between the study conducted in Egypt and the present study can be due to racial, ethnic, and cultural differences that affect the level of the expectations of the people. A study conducted in Turkey on 102 infertile couples showed that the share of sexual dissatisfaction for men and women was respectively 37.3 and 33.3% (20). In comparing the results of studies in the area of sexual satisfaction, characteristics of the research subjects should be considered. The findings from 64 papers showed that infertility is one of the factors influencing sexual satisfaction (37).

**Table 3:** Results of Chi-square and Cramer’s V tests to examine the relationship between health literacy, sexual function, and sexual satisfaction in infertile couples referred to the Royan Institute in 2016


Variable	Sample	Sexual function			Sexual satisfaction		
		df	P value	Cramer’s V	df	P value	Cramer’s V

Health literacy	Woman	2	0.005	0.33	6	0.007	0.17
	Man	8	0.017	0.18	6	0.038	0.16


df; Degree of freedom.

A significant relationship between health literacy and sexual function and between health literacy and sexual satisfaction was observed in this study. Health literacy affects attitude, mental condition, behaviors related to health, and thus, physical health status. In addition, health literacy can affect the use of information and sexual function in couples (38), and, thereby have a positive impact on quality of life (39). A study of 290 women by Mogadam Banaem et al. (40) showed that the rate of sexual satisfaction is related to health literacy level. In this regard, it can be stated that health literacy can affect mental states and attitude of the couples to sexual and marital relations, and thus, they can affect their sexual satisfaction.

Although the research has reached its aims, there were some unavoidable limitations. First, this study was cross-sectional and conducted in one center. Second, research variables are dependent on the socio-cultural environment. Therefore, to generalize the results for large groups in different settings, it is recommended that studies be performed in other places and with a larger sample. Also, it can be recommended to conduct a meta- analysis of existing literature to further understanding in this field.

## Conclusion

The results of this study showed that marginal health literacy was more common than adequate or inadequate health literacy in both sexes. Additionally, our study confirmed the relationship between health literacy and sexual function and sexual satisfaction. Accordingly, it is recommended that applied and practical plans be developed in order to improve health literacy at the level of the community, especially among infertile couples. 
